# Control of corruption, democratic accountability, and effectiveness of HIV/AIDS official development assistance

**DOI:** 10.3402/gha.v9.30306

**Published:** 2016-05-13

**Authors:** Hwa-Young Lee, Bong-Ming Yang, Minah Kang

**Affiliations:** 1 LEE Jong-Wook Center for Global Medicine, College of Medicine, Seoul National University, Seoul, Republic of Korea; 2 Graduate School of Public Health, Seoul National University, Seoul, Republic of Korea; 3 Department of Public Administration, College of Social Sciences, Ewha Womans University, Seoul, Republic of Korea

**Keywords:** HIV/AIDS aid, governance, official development assistance, aid effectiveness, control of corruption, democratic accountability

## Abstract

**Background:**

Despite continued global efforts, HIV/AIDS outcomes in developing countries have not made much progress. Poor governance in recipient countries is often seen as one of the reasons for ineffectiveness of aid efforts to achieve stated objectives and desired outcomes.

**Objective:**

This study examines the impact of two important dimensions of governance – control of corruption and democratic accountability – on the effectiveness of HIV/AIDS official development assistance.

**Design:**

An empirical analysis using dynamic panel Generalized Method of Moments estimation was conducted on 2001–2010 datasets.

**Results:**

Control of corruption and democratic accountability revealed an independent effect and interaction with the amount of HIV/AIDS aid on incidence of HIV/AIDS, respectively, while none of the two governance variables had a significant effect on HIV/AIDS prevalence. Specifically, in countries with accountability level below −2.269, aid has a detrimental effect on incidence of HIV/AIDS.

**Conclusion:**

The study findings suggest that aid programs need to be preceded or at least accompanied by serious efforts to improve governance in recipient countries and that democratic accountability ought to receive more critical attention.

## Introduction

The HIV/AIDS epidemic has been a serious threat to the world that deserves global attention. World Health Organization (WHO) statistics show that by 2013, 35 million people were living with HIV and 1.5 million people have died of AIDS (1). More than 95% of HIV infections reside in developing countries. Over a decade, global communities have invested tremendous resources on HIV/AIDS. For example, financial aid from donor countries increased almost seven times from USD1.2 billion in 2002 to USD8.5 billion in 2013 (2). In addition, not only did aid donor communities with large funds newly emerge (e.g., Bill & Melinda Gates Foundation), creative aid mode (e.g., budget support using a sector-wide approach) has also been continuously conceived in the international development community.

Despite all these global efforts, however, HIV/AIDS outcomes in developing countries have regrettably not shown significant progress. Specifically, AIDS-related deaths showed no significant decline, decreasing from 2.1 million in 2002 to 1.8 million in 2011, and people living with HIV even rose from around 31 million in 2002 to 34 million in 2011 (3). This critical situation certainly causes concern for aid effectiveness among international development communities and has led to warranted attention among researchers to investigate what the determinants for aid effectiveness are.

The World Bank, for example, claims governance of recipient countries is one of the foremost reasons why official development assistance (ODA) efforts have been largely ineffective; this claim is supported by academia and global development agencies (4, 5). Many researchers like Santiso (6) and Burnside and Dollar (7) have tried to demonstrate a visible relationship between governance of recipient countries and outcomes of ODA by quantitative analyses. Although the results of these studies were divergent, the wide belief that aid efforts have more potential for success in well-governed countries has influenced donors’ policies to the extent that some donors created an aid program for improvement of governance itself or attached good governance as a precondition for disbursing assistance (8).

As in Kaufmann's article (9), control of corruption and accountability are the most commonly cited governance factors in aid effectiveness discussion among several dimensions. Similarly, Lewis (10) also asserted that accountability, government effectiveness, and control of corruption are the most relevant to healthcare delivery. This is even more true in the case of HIV/AIDS aid effectiveness, as HIV/AIDS control usually requires a huge amount of funding due to the scale of the epidemic, high price of treatment medicines, as well as the social stigma attached to it. Therefore, the scale of financial resources invested into it provides extended chances and the scope of corruption in HIV/AIDS contexts more than any other diseases (10). In many instances, corruption siphons off public revenue, raising the price of services for HIV/AIDS prevention and treatment while deteriorating quality and quantity of service (11). This not only deteriorates ODA outcomes, but also makes people reluctant to co-pay for services, which in turn reduces public revenue, restricting the government's capacity to provide quality public services. The vicious cycle discourages donor countries from wanting to allocate further aid.

Corruption is seen as a pervasive issue, as corruptive decisions and behaviors can be committed by anyone at all levels, from high officials down to service providers at the lowest level of money flow. Even when assistance is provided by non-governmental actors bypassing recipient government, there is still room for corrupt behavior that influences aid effectiveness.

Democratic accountability is yet another important governance factor in HIV/AIDS control. Because HIV/AIDS is sexually transmitted and society attaches a social stigma to it, HIV/AIDS response efforts require a multi-faceted approach that involves dedicated human resources and sustained policy commitment (12, 13). Also, due to its scale and complexity of response, HIV/AIDS programs entail a kind of trade-off such as downsizing or sometimes abandonment of other public priorities (14). All these give rise to difficult and debatable public policy choices at national and international levels. Consequently, if HIV/AIDS policies established without broad agreement via a democratic process, they can be fragile when political environments change (15).

Democratic accountability can be realized through various means: fair election, guaranteed freedom of mass media, and active civil society (16). A fair election process can expose the government's activities for HIV/AIDS control to the population transparently and serve as an incentive for national leaders to cope with the problems properly. In addition, democratically elected governments are more likely to gain public trust leading to public acceptance of HIV/AIDS-related measures (12). Similarly, mass media with freedom of speech can monitor governmental activities for HIV/AIDS more strictly by revealing government's ‘misgovernance’ such as incompetence, negligence, corruption, ineffectiveness, risky behavior, and so on. An active civil society can enlarge the funding scale, which enables more comprehensive and diverse actions for HIV/AIDS, and also create social capital such as interpersonal trust, which can supplement a government's role on social welfare or social services for HIV/AIDS patients (15).

Despite emphasis on relevance of governance with health outcomes including HIV/AIDS from a theoretical perspective, however, empirical evidence supporting it is lacking, and even the results of existing evidences are not consistent. For example, based on two stage least square (TSLS) analyses, Gupta et al. (17) showed a negative relationship between the level of control of corruption and infant/child mortality and low birth weight. Rajkumar and Swaroop (18) also demonstrated that public expenditure and control of corruption had a significant negative interaction on child mortality, meaning that public expenditure on health decreases under-five child mortality rate more in less corrupt countries. On the other hand, Dietrich's study (19) presented findings contrary to the above studies and showed that the more corrupt countries were, the stronger the relationship between volume of health aid and DPT vaccination completion rate was. According to Dietrich, in a move to pursue rent-seeking in more lucrative areas, corrupt governments were likely to implement aid effectively in sectors where compliance costs were relatively low such as public health. There is only one study when we narrow the scope of health outcome down to HIV/AIDS, to our knowledge. Bassolé (20) demonstrated through OLS regression that governance level measured by Worldwide Governance Indicator (WGI) were negatively associated with HIV/AIDS prevalence. Thus, in an attempt to fill this gap, this study aims to explore possible causal relationships between the two governance factors of aid recipient countries and effectiveness of HIV/AIDS ODA.

## Methods

### Measures and data source

#### Dependent variables

Prevalence and incidence of HIV/AIDS were selected for measuring HIV/AIDS aid effectiveness because they can capture the effect from all types of HIV/AIDS programs while intermediate outcomes, e.g., rate of condom usage or anti-retroviral treatment coverage, reflect effect from only particular types of programs. The unit and data source of HIV/AIDS prevalence and incidence are presented in Table 1.

**Table 1:** Data sources

Variables	Source / unit
**Dependent variables**	
HIV/AIDS prevalence	World Bank / % of people with HV/AIS among population ages 15–49
HIV/AIDS incidence	UNAIDS Report on the Global AIDS Epidemic (2012) / % of newly infected persons among population ages 15–49
**Independent variables**	
Corruption/accountability	World Bank / Range from −2.5 to 2.5. Higher score means better governance
HIV/AIDS disbursement	Institute for Health Metrics and Evaluation (IHME) / HIV/AIDS disbursement (public/multi) per capita ($)
Ethnic fractionalization	Alesina et al. (2003) / Range from 0 to 1. 1 means racially homogenous nation
Proportion of Muslim	Pew Research Center / Muslim proportion of all population %
GNI per capita	World Bank / GNI converted to international dollars by PPP§ rates ($)
Gender economic inequality	World Bank / % of girls to boys in primary and secondary education in schools
Share of internet user	World Bank / The number of people with access to the worldwide network per 100
Share of health expenditures	World Bank / % of total public and private health expenditure of GDP
Human right protection	Cingranelli-Richards (CIRI) / Range from 0 to 30, Higher score means better human right guarantee

§ PPP=purchase parity power.

#### Governance variables

‘Control of corruption’ and ‘voice and accountability’ which are sub-dimensions of WGI complied by the World Bank were used as indicators for level of corruption and democratic accountability, respectively. ‘Control of corruption’ measures the perception of how much public power is exercised for private gain, including both petty and grand forms of corruption, and how much the state is captured by elites and private interests (9). As an index for democratic accountability, ‘voice and accountability’ of the WGI was also used. It measures the perception of the extent to which citizens are able to participate in selecting their own government as well as of freedom of expression and of mass media (9). Both scores range from −2.5 to 2.5. A higher score indicates better control of corruption and higher level of democratic accountability.

#### HIV/AIDS disbursement

Public and multinational HIV/AIDS disbursement per capita, which was compiled by the Institute for Health Metrics and Evaluation, was summed and converted to 2010 constant USD (21).

#### Other control variables

The factors that previous studies had shown to be associated with HIV/AIDS outcomes theoretically and empirically were chosen as control variables. Muslims are strictly prohibited from extramarital sexual relationship and alcohol consumption and are highly likely to undergo circumcision, all of which are expected to be suppressing factors for spread of HIV/AIDS (22). Ethnicity is also related to HIV/AIDS outcomes. A country with a high level of ethnic fractionalization has more frequent conflicts. This impedes policy-making and economic development, which may in turn have a negative impact on HIV/AIDS outcomes (23).

The level of economic development is widely recognized as one of the important factors associated with health outcomes. For example, high rates of unemployment and low wages increase the number of the population working in the sex industry and this raises the likelihood of HIV/AIDS infection (24). Gross national income (GNI) per capita was used as an indicator for this. Gender inequality is also a factor proved to be associated with HIV/AIDs outcomes. Because women faced with economic vulnerability are more likely to work in the sex industry than the men in a similar situation, economic inequality unfavorable for women increases supply of sex workers. This raises the chances for women to be exposed to HIV/AIDS infection (25). Due to unavailability of data on income gaps between males and females in developing countries, differences in school enrollments were used as a proxy variable based on the positive relationship between education and income. Lack of information about safe sexual culture and inadequate knowledge of refusal techniques against demands on risky sexual activity increase the probability of HIV/AIDS infection (25). The proportion of internet users was used as an indicator for accessibility to information about safe sexual culture and refusal skill against risky sexual activity. Lastly, national healthcare expenditure was included as a covariate in the model because it has an effect on overall health status of the population. The data sources and units of each variable are listed in Table 1.

### Statistical analysis

**Fig. 1:**
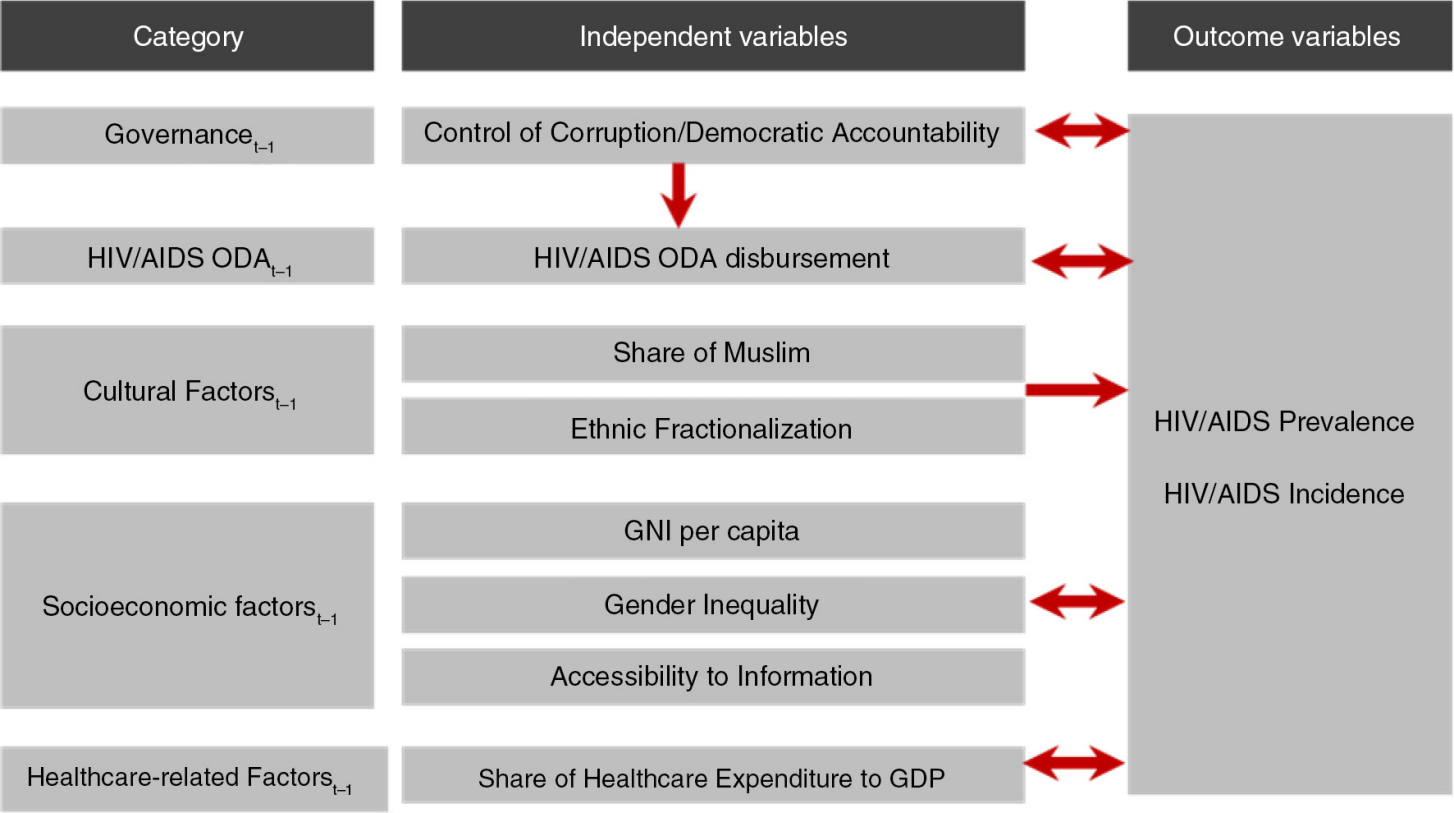
Analysis framework.

Figure 1 presents the conceptual framework for the analysis. Several independent variables and incidence of HIV/AIDS were log-transformed based on variance and skewness of data. HIV/AIDS prevalence was logit-transformed considering the S-shaped distribution of the data (20, 24, 25).

An OLS regression was performed as an exploratory analysis although results from OLS regression are expected to be biased due to the following reasons. First, there is a possibility of endogeneity occurring by ‘simultaneity’ between some of the explanatory variables (e.g., socio-economic status) and outcome variables (26). For example, good socio-economic status may improve HIV/AIDS outcomes while HIV/AIDS disease may limit working and earning capacity of patients, lowering their socio-economic status. Recent trends, where development aid is disbursed based on earlier performances of recipient countries can even raise the chance of simultaneity (27, 28). Potential measurement errors may also be cause for bias. Some donor countries might exaggerate the amount of aid they donate and this measurement error is not related to characteristics of the recipient countries (29). Lastly, as in other analyses, the problem of ‘omitted variable’ might arise since it is hard to ensure whether all possible ways in which countries might differ are well-controlled in cross-country regressions (29).

What has been most commonly used in previous studies for addressing these biases is TSLS with instrumental variable (IV) (7, 30, 31). However, dynamic panel Generalized Method of Moments (GMM) estimation with country-fixed effect was undertaken instead of TSLS in our study because there is an ongoing controversy on exogeneity of IVs (29, 32). Other covariates with long-lasting nature such as country's health system or institutions, which might affect aid effectiveness, are controlled for country-fixed effect. The regression where countries are indexed by ‘r’ and time by ‘t’ can be formulated as:

**Table:** 

InOutcome_rt_ = *α*InOutcome_rt-1_+*β*InF_rt-1_+*γ*G_rt-1_+*δ*InF_rt-1_·G_rt-1_+*θ*X_rt-1_+s_r_+*ε*_rt_	

•	Outcome_rt-1_: Outcome of r Country in the previous year
•	F_rt-1_: Disbursement per capita for HIV/AIDS in r Country in the previous year
•	G_rt-1_: Governance index of r Country in the previous year
•	X_rt-1_: Other control variables in r Country in the previous year
•	S_r_: r country-fixed effect


The unit of analysis is country-year. All of the explanatory variables were lagged to capture time gap in which explanatory factors take effect on the outcome (19). To measure conditional effect of governance on HIV/AIDS aid effectiveness, interaction terms of amount of disbursement for HIV/AIDS and governance variables were employed (7, 18).

Generally, for an over-identified model like this, GMM is known to be more effective and hence has been used more commonly (33). Among two methods of GMM, system-GMM estimation was employed rather than ‘difference-GMM estimation’ in our study as it is recognized as more precise and efficient (34). Once GMM estimators were obtained, joint validity test of the instruments is standard procedure (35). First, validity of over-identification restriction was verified with the Sargan-test that examined the null hypothesis, ‘all instruments chosen should not be correlated with residuals’. When this hypothesis is not rejected, the validation of instruments is obtained. It is necessary to note that rejected hypothesis does not necessarily mean that over-identification is not appropriate. Autocorrelation was also tested. Second-order autocorrelation of Δe_rt_ should not be allowed in GMM estimation while first-order autocorrelation can be.

Sensitivity analyses were performed to test the robustness of the results. Firstly, data were re-analyzed after outliers were removed. For this, ‘Dfbetas’ of lagged prevalence and incidence were calculated and outliers were defined as observations with Dfbetas bigger than $2/\sqrt{n}$. Secondly, missing data were inputted using Markov Chain Monte Carlo (MCMC) multiple imputation method which was considered to be the best fit for the missing pattern of this dataset (36). All the analyses were performed with StataSE 11.2.

## Results

Table 2 shows descriptive statistics of the dataset used for analysis. Data for dependent variable cover years 2001–2010, while those for independent variables are between 2000 and 2009 due to a year lag between the dependent variables and independent variables.

**Table 2:** Descriptive statistics for study sample

	Obs.	Mean	Std. Dev.	Obs.	Mean	Std. Dev.
		
Explanatory variables	Prevalence dataset			Incidence dataset		
HIV/AIDS prevalence/incidence	333	3.87	6.55	212	0.57	0.90
Voice and accountability	333	−0.39	0.69	212	−0.44	0.62
Corruption	333	−0.47	0.58	212	−0.53	0.52
Aid ($)	333	2.56	7.99	212	3.43	9.61
Enroll ratio (%)	333	91.95	12.18	212	91.44	11.89
Ethnic fractionalization	333	0.55	0.24	212	0.59	0.22
GNI per capita ($)	333	3,412.88	3,550.10	212	3,052.78	3,655.99
Health expenditure/GDP (%)	333	5.97	1.70	212	5.99	1.81
Share of internet user (%)	333	5.83	8.34	212	4.64	7.63
Share of Muslim (%)	333	25.50	35.52	212	27.22	33.54

Average HIV/AIDS prevalence of countries in the 2001–2011 dataset was 3.87%, which is quite high when compared to the global average during the same period (by approximately 0.8%). The average governance scores were lower as well (Table 2). About USD2.56–3.43 per capita were supported for HIV/AIDS control through ODA. This is largely because the analysis involved developing countries that have HIV/AIDS ODA recipient country status. Average scores of control of corruption and democratic accountability were −0.47 and −0.39, respectively.

The result of dynamic panel analysis differed from that of OLS analysis (Tables 3 and 4). Unlike its significant independent association with HIV/AIDS prevalence shown in the OLS analysis, control of corruption showed neither independent effect nor interaction with amount of assistance on HIV/AIDS prevalence in GMM estimation. Similarly, control of corruption had only independent and negative effect on HIV/AIDS incidence at significance level of 0.01 in GMM estimation while it showed interaction with amount of assistance on incidence of HIV/AIDS in OLS analysis.

**Table 3:** Results from OLS analysis for relationship between governance and HIV/AIDs outcomes

		HIV/AIDS prevalence		HIV/AIDS incidence	
			
		(1)	(2)	(3)	(4)
**Control of corruption**	Lagged prevalence (logit)	*0.974	*0.974		
	Lagged incidence (ln)			*1.026	*1.028
	Aid (ln)	0.006	0.007	*−0.038	*−0.033
	Enroll ratio	0.001	0.001	0.001	0.001
	Ethnic fractionalization	0.003	0.001	−0.078	−0.082
	GNI per capita (ln)	0.008	0.003	−0.011	−0.021
	Health expenditure	0.002	0.002	*0.016	*0.017
	Share of internet user (ln)	−0.005	−0.003	*0.038	*0.042
	Share of Muslim	0.001	0.001	*0.021	*0.020
	Control of corruption	**−0.041	**−0.040	**−0.052	−0.013
	Aid (ln) * Control of corruption		0.006		**0.024
	Constant	−0.275	−0.247	−0.162	−0.119
**Accountability**	Lagged prevalence (logit)	*0.973	*0.973		
	Lagged incidence (ln)			*1.021	*1.021
	Aid (ln)	0.005	0.005	*−0.040	*−0.041
	Enroll ratio	0.001	0.001	0.001	0.001
	Ethnic fractionalization	0.027	0.023	−0.047	−0.041
	GNI per capita (ln)	0.006	0.004	−0.026	−0.024
	Health expenditure	0.004	0.004	**0.014	**0.014
	Share of internet user (ln)	−0.001	0.000	*0.042	*0.041
	Share of Muslim	0.000	0.000	**0.018	**0.018
	Accountability	*−0.048	*−0.047	−0.022	−0.031
	Aid (ln) * Accountability		0.003		−0.004
	Constant	−0.310	−0.292	−0.048	−0.067

* *p*<0.05

** *p*<0.01.

**Table 4:** Results from a panel System-GMM analysis for effect of governance on HIV/AIDs outcomes

Dependent variables		HIV/AIDS prevalence		HIV/AIDS incidence	

Model		(1)	(2)	(3)	(4)
**Control of corruption**	Lagged prevalence (logit)	*0.938	*0.942		
	Lagged incidence (ln)			*0.924	*0.930
	Enroll ratio	0.004	0.003	−0.008	−0.008
	GNI per capita (ln)	0.069	0.081	−0.031	−0.028
	Aid (ln)	0.009	−0.002	* − 0.066	* − 0.066
	Health expenditure	0.013	0.011	−0.016	−0.016
	Share of internet user (ln)	−0.012	−0.020	*0.077	*0.076
	Control of corruption	0.064	0.079	* − 0.238	* − 0.235
	Aid (ln) * Control of corruption		−0.032		0.002
	Constant	−1.143	−1.114	0.767	0.730
Sargan-Hansen test		0.0006	0.0011	0.0016	0.0017
A-R (1): Serial correlation test		0.0353	0.0326	0.0089	0.0098
A-R (2): Serial correlation test		0.8468	0.8699	0.6310	0.6272
No. of instrument (No. of group)		32 (56)	33 (56)	36 (34)	37 (34)
No. of observations		219	219	142	142
**Accountability**	Lagged prevalence (logit)	*0.938	*0.942		
	Lagged incidence (ln)			*0.924	*0.913
	Enroll ratio	0.003	0.003	−0.004	−0.005
	GNI per capita (ln)	0.082	0.087	0.001	−0.022
	Aid (ln)	0.012	0.005	* − 0.094	* − 0.114
	Health expenditure	0.010	0.010	−0.002	−0.004
	Share of internet user (ln)	−0.011	−0.014	**0.065	**0.067
	Accountability	0.066	0.065	* − 0.242	* − 0.192
	Aid (ln) * Accountability		−0.021		** − 0.052
	Constant	−1.145	−1.111	0.047	0.411
Sargan-Hansen test		0.0007	0.0010	0.0012	0.0015
A-R (1): Serial correlation test		0.0316	0.0295	0.0104	0.0220
A-R (2): Serial correlation test		0.6039	0.4061	0.5312	0.5270
No. of instrument (No. of group)		32 (56)	33 (56)	36 (34)	37 (34)
No. of observations		219	219	142	142

* *p*<0.05

** *p*<0.01.

**Fig. 2:**
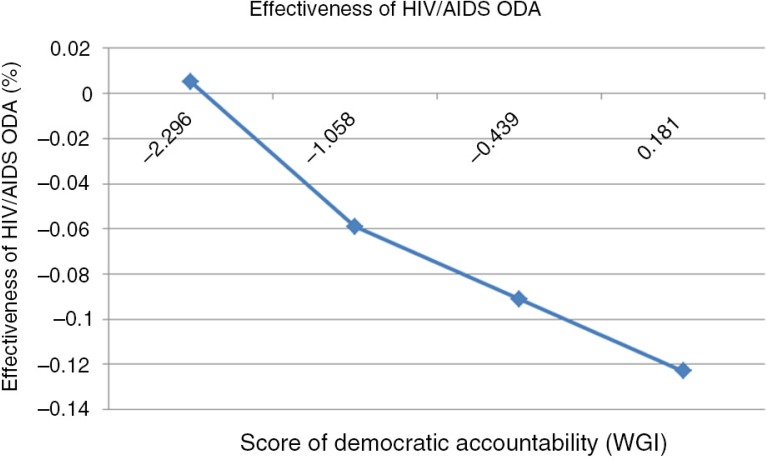
Effectiveness of ODA for HIV/AIDS according to the level of democratic accountability.

The democratic accountability variable also showed the same pattern. While there were significant negative associations between the level of accountability and HIV/AIDS prevalence in the OLS analysis, none of the independent variables, including democratic accountability, were significant on HIV/AIDS prevalence in the GMM estimation. However, in terms of predicting the HIV/AIDS incidence, significant interaction between democratic accountability and amount of HIV/AIDS assistance was found in the GMM estimation. Figure 2 presents changes in aid effectiveness on HIV/AIDS incidence with varying levels of accountability.^1^

At the average level of accountability (WGI score: −0.439), more assistance money per capita can be linked to lower HIV/AIDS incidence. However, this trend turns in the opposite direction when the level of democratic accountability reaches a turning point, which is −2.120 based on the WGI accountability index. When the level of democratic accountability is 2STD subtracted from the average (WGI score: −2.269), HIV/AIDS incidence rose as the amount of assistance per capita increased. The results from the sensitivity analyses were not different as in the main analysis, except for a small change in the significance level (Appendix Table 1).

## Discussion

This paper analyzed the effect of control of corruption and democratic accountability on relationship between ODA amount and prevalence or incidence of HIV/AIDS in ODA recipient countries. Guided by economic theory, we specified a dynamic panel data model where two governance dimensions – control of corruption and democratic accountability – had significant independent or moderating effect on HIV/AIDS incidence, respectively, while on prevalence of HIV/AIDS, neither of them had a significant effect. The result may come from the ongoing emphasis on ODA funding for the HIV-AIDS preventive programs. Although a large proportion of HIV funding shifted from prevention toward the treatment and care since 1990, a prevention-focused program has still prioritized during the 2000s (37). Since the biggest risk factor for HIV/AIDS infection is unsafe sex, prevention strategy such as education on sexual behavior is very effective (38). Prevention program deters the occurrence of new patients, decreasing incidence rate of HIV/AIDS. However, a decrease in the total number of HIV-AIDS patients in a mere one-year time span is difficult because a prevention program needs a longer time span to lower the prevalence. In addition, treatment can delay the progression of disease, thereby prolonging the lifespan of existing HIV/AIDS patients, while it does not completely cure the disease. In such cases, treatment programs may increase the prevalence of HIV/AIDS by reducing the death rate of HIV/AIDs patients. For these reasons, we should be cautious not to interpret the non-significance on the prevalence as aid futility (39).

In our study, the two governance dimensions showed different patterns in predicting the HIV/AIDS outcomes. While control of corruption had only independent effect on HIV/AIDS incidence, meaning that it does not have a boosting or aggravating effect on aid effectiveness, democratic accountability showed a negative interaction with amount of HIV/AIDS assistance on HIV/AIDS incidence. Such an additive effect on reducing the HIV/AIDS incidence means that aid would be more effective if it was conditioned on good democratic accountability. It is assumed that due to the nature of corruption that it is committed ‘in the dark’, improvement in the control of corruption is not easily noticed by others. A decrease in the level of corruption therefore may not have a motivational effect on other dimensions of governance. On the contrary, accountability, defined as ‘the responsibility to be able to show that they have fulfilled their original responsibility’ is a clearly visible concept (40). For this reason, performance of high democratic accountability is readily highlighted.

Findings of our study showed that in a country with accountability level below a certain level (e.g., −2.269 in WGI score), aid has a detrimental effect on incidence of HIV/AIDS. On the other hand, in countries with scores of above −2.269, additional aid seemed to decrease the incidence of HIV/AIDS. This is in line with the results of Kosack (41) and Knack (42) where in countries with bad governance, more aid not only increases their dependency on aid but also increases chances for corruption, thus lowering its overall performances.

Finally, our study yielded different results when we adopted OLS and dynamic panel analysis. This may mean that the presence of endogeneity and country-fixed effect in the relationship between explanatory variables and HIV/AIDS outcomes cannot be regarded as inconsequential. Finding that aid can be rather harmful on HIV/AIDS incidence in countries with low levels of democratic accountability is reaffirming the view that aid programs for control of HIV/AIDS must be preceded or at least accompanied by an effort for enhancing democratic accountability of recipient country.

Findings of our study should be interpreted with consideration of the following limitations. First, because of the missing values in each variable, the number of countries that remained in the final dataset for analysis reduced significantly. Although the robustness of the results was checked through imputation of missing values, our results could not be interpreted in the same rigorous way as results that we would have from the analysis using a full dataset. Second, while our model controlled the simultaneity and country-fixed effect, there might still be remaining time-variant factors we could not identify. Therefore, focusing on the size of coefficients would be meaningless, while result of this study can be valuable in gaining an understanding of general trends and directions of variables. Third, even if our findings suggest positive effect of the two governance factors on HIV/AIDS control efforts with statistical significance in a quantifiable measure, it is also very important to look at the effect of two governance factors in each country under the individual country's specific context such as socio-economic, cultural, and political context. Qualitative studies can fill this gap and we hope that our research can be a starting point to initiate such mixed-method research in the future.

## Conclusion

In spite of the positive impact of control of corruption and democratic accountability on effectiveness of ODA as suggested in this study, it is disappointing to note that control of corruption and democratic accountability in the 56 countries has largely deteriorated since 2000 (World Bank data).^2^ This can be deemed as one of the reasons why the HIV/AIDS epidemic is still at a worrying level despite continuously increasing global attention and resource investments (43). Nevertheless, it is expected that international funding will continue to be a valuable resource for the fight against HIV/AIDS in the future. Without considering governance factors, however, questions and issues of aid effectiveness would not easily be addressed and continue to haunt over relevant actors incessantly. Presumably, some countries may show effective performance in spite of weak governance. Our analysis implies that even these countries with good progress based on weak governance would attain far better outcomes if they improve their governance. Considering all this, support to improve the governance in recipient countries, especially democratic accountability, should be made together with HIV/AIDS aid. This kind of support is being adopted in some countries, in the form of support for political democratization or economic liberalization (44).

Overall, it is noteworthy that future strategy for HIV/AIDS control should be planned under the careful consideration of each individual country. It is hoped that this article can be a starting point for such future research efforts.

## Footnotes

1 $\frac{\%\Delta \text{outcome}}{\%\Delta \text{HIV}/\text{AIDS aid}}=\beta +\delta \cdot {\text{G}}_{\text{r}}$ (β: coefficient of aid variable, δ: coefficient of interaction term, G_r_: governance index)

2 www.info.worldbank.org/governance/wgi/index.aspx#home

## Paper context

Very few studies have previously addressed the association between recipient country's governance and HIV/AIDS outcomes or moderating effect of governance in the aid-effectiveness. Our study provides new evidence that control of corruption and democratic accountability in recipient country have direct and moderating effect on HIV/AIDS control, respectively. The study findings suggest that HIV/AIDS programs need to be accompanied by efforts to improve governance in recipient countries.

## Appendix

**Appendix Table 1:** Results of sensitivity analyses

	HIV prevalence				HIV incidence			
		
Dependent variable	Control of corruption		Accountability		Control of corruption		Accountability	
			
Model	(1)	(2)	(3)	(4)	(5)	(6)	(7)	(8)
**Elimination of outliers**								
Lagged prevalence (logit)	*0.961	*0.961	*0.957	*0.954				
Lagged incidence (ln)					*1.070	*1.052	1.017	0.998
Enroll ratio	0.006	0.006	0.007	0.007	−0.008	−0.009	−0.002	−0.003
GNI per capita (ln)	−0.056	−0.057	−0.069	−0.074	0.193	0.217	0.086	0.110
Aid (ln)	0.001	0.001	0.002	0.005	** − 0.039	* − 0.047	* − 0.064	* − 0.076
Health expenditure	0.009	0.010	0.009	0.010	−0.014	−0.014	0.000	−0.002
Share of internet user (ln)	−0.009	−0.008	−0.007	−0.005	**0.048	**0.042	*0.047	**0.040
Control of corruption/accountability	−0.029	−0.031	−0.006	−0.015	* − 0.253	* − 0.299	* − 0.267	* − 0.344
Aid (ln) * Control of corruption/account		0.002		0.010		−0.030		** − 0.052
constant	−0.365	−0.364	−0.293	−0.274	−0.730	−0.854	−0.621	−0.678
No. of observations	196	196	196	196	128	128	128	128
**Imputation of the missing**								
Lagged prevalence (logit)	*0.961	*0.961	*0.957	*0.954				
Lagged incidence (ln)					*1.070	*1.052	1.017	0.998
Enroll ratio	0.006	0.006	0.007	0.007	−0.008	−0.009	−0.002	−0.003
GNI per capita (ln)	−0.056	−0.057	−0.069	−0.074	0.193	0.217	0.086	0.110
Aid (ln)	0.001	0.001	0.002	0.005	** − 0.039	* − 0.047	* − 0.064	* − 0.076
Health expenditure	0.009	0.010	0.009	0.010	−0.014	−0.014	0.000	−0.002
Share of internet user (ln)	−0.009	−0.008	−0.007	−0.005	**0.048	**0.042	*0.047	**0.040
Control of corruption/accountability	−0.029	−0.031	−0.006	−0.015	* − 0.253	* − 0.299	* − 0.267	* − 0.344
Aid (ln) * Control of corruption/account		0.002		0.010		−0.030		** − 0.052
constant	−0.365	−0.364	−0.293	−0.274	−0.730	−0.854	−0.621	−0.678
No. of observations	196	196	196	196	128	128	128	128

* *p*<0.05

** *p*<0.01.
